# Analysis of IL-17A, IL-17F, and miR-146a-5p Prior to Transplantation and Their Role in Kidney Transplant Recipients

**DOI:** 10.3390/jcm13102920

**Published:** 2024-05-15

**Authors:** Barbara Wysoczańska, Marta Dratwa, Artur Nieszporek, Wanda Niepiekło-Miniewska, Dorota Kamińska, Tomasz Ramuś, Julia Rasała, Magdalena Krajewska, Katarzyna Bogunia-Kubik

**Affiliations:** 1 Laboratory of Clinical Immunogenetics and Pharmacogenetics, Hirszfeld Institute of Immunology and Experimental Therapy, Polish Academy of Sciences, 53-114 Wroclaw, Poland; marta.dratwa@hirszfeld.pl (M.D.); katarzyna.bogunia-kubik@hirszfeld.pl (K.B.-K.); 2 Biobank Research Group, Lukasiewicz Research Network—PORT Polish Center for Technology Development, 54-066 Wroclaw, Poland; 3 Laboratory of Tissue Immunology, Medical Center, Hirszfeld Institute of Immunology and Experimental Therapy, Polish Academy of Sciences, 53-114 Wroclaw, Poland; wanda.niepieklo-miniewska@hirszfeld.pl; 4 Department of Nephrology and Transplantation Medicine, Wroclaw Medical University, 50-367 Wroclaw, Poland; dorota.kaminska@umw.edu.pl (D.K.); magdalena.krajewska@umw.edu.pl (M.K.); 5 Faculty of Medicine, Wroclaw Medical University, 50-367 Wroclaw, Poland; tomasz.ramus@student.umw.edu.pl; 6 Milicz Medical Center, 56-300 Milicz, Poland; julia.rasala@gmail.com

**Keywords:** kidney transplantation outcome, kidney recipients, IL-17A/F, miR-146a-5p

## Abstract

**Background/Objectives**: The balance between regulatory and Th17 cells plays an important role in maintaining the immune tolerance after kidney transplantation (KTx) which is essential for transplantation success, defined as a long graft survival and an absence of organ rejection. The present study aimed to assess whether the pretransplant characteristics of IL-17A and IL-17F, their receptors, as well as miR-146a-5p, an miRNA associated with IL-17A/F regulation, can predict KTx outcomes. **Methods**: A group of 108 pre-KTx dialysis patients and 125 healthy controls were investigated for single nucleotide substitutions within genes coding for *IL-17A*, *IL-17F*, their *IL-17RA*/*RC* receptors, and *miR-146a-5p*. Genotyping was performed using LightSNiP assays. In addition, IL17-A/F serum concentrations were determined using ELISA while miR-146a-5p expression was analyzed by RT-PCR. **Results**: The *IL-17F* (rs763780) *G* allele prevailed in KTx recipients as compared to healthy individuals (OR = 23.59, *p* < 0.0001) and was associated with a higher IL-17F serum level (*p* = 0.0381) prior to transplantation. Higher miR-146a-5p expression before KTx was more frequently detected in recipients with an increased IL-17A serum concentration (*p* = 0.0177). Moreover, *IL-17A* (rs2275913) *GG* homozygosity was found to be associated with an increased incidence of deaths before KTx (OR = 4.17, *p* = 0.0307). T-cell or acute rejection episodes were more frequently observed among patients with the *C* allele of *miR-146a-5p* (rs2910164) (OR = 5.38, *p* = 0.0531). *IL17-RA/-RC* genetic variants (*p* < 0.05) seem to be associated with eGFR values. **Conclusions**: These results imply that *IL-17F* (rs763780) polymorphism is associated with the serum level of this cytokine and may be related to the risk of renal disease and transplant rejection together with *miR-146a-5p* (rs2910164), while the *IL-17A* (rs2275913) genotype may affect patients’ survival before KTx.

## 1. Introduction

Kidney transplantation (KTx) is a treatment option for patients with end-stage renal disease (ESRD). It significantly improves the patient’s quality of life, also leading to the transplant’s substantial prolongation [1,2].The long-term allograft as well as the recipient survival after KTx determine the success of the transplant procedure. New immunological and genetic biomarkers associated with transplantation success, a reduced risk of death and complications are still being investigated.

It is important that potential biomarkers demonstrate practical usefulness in monitoring the course of transplantation and, at the same time, differentiate between the parameters indicating early or late renal dysfunction after transplantation [3]. Currently, the basic parameters associated with donor–recipient HLA incompatibility, or the presence of preformed or de novo anti-HLA antibodies, constitute the main diagnostic, prognostic and therapeutic biomarkers [4,5,6]. Understanding the activation or suppression of specific subpopulations of T cells in kidney transplant recipients and elucidating the role of the signaling pathways involved in the production of specific cytokine profiles is helpful in determining the recipient’s involvement in the progression of donor cell acceptance or graft rejection [7,8]. Many studies show the significance of the balance between Th17 and T regulatory (Treg) cells together with their profile of inflammatory cytokines and their receptors for the transplantation outcome [9,10,11]. The Th17/Treg ratio is essential for maintaining the balance and homeostasis of the immune response. A reduced Th17/Treg ratio may induce immune tolerance and prolong allograft survival, while an increased ratio may lead to its rejection [12,13]. Additionally, increasing evidence indicates that Treg cells may be involved in various kidney diseases. Treg lymphocytes may play a negative regulatory role in kidney diseases and inhibit the immune response through direct cell contact or the secretion of inhibitory cytokines [14]. At the same time, disease processes within the kidney may in turn affect the function of Treg cells. For example, the number of Treg cells in patients with IgA nephritis is significantly reduced [15].

Viral infections continue to be a significant cause of morbidity and mortality after KTx [16]. Understanding the role of Th17 in the context of viral infections may improve the prediction of clinical outcomes and patient treatment [17]. Recent studies have demonstrated the effector functions of Th17 cells in the host immune response to viruses, including their key role in the production of pro-inflammatory cytokines and the activation of other immune cells [18]. Some Th17 cells can also modulate the immune response and secrete immunosuppressive factors such as IL-10. Sadeghi et al. demonstrated that the suppression of IL-17 production promotes chronic polyomavirus BK (BKV) and may increase viral replication [19]. In KTx recipients, BKV causes polyomavirus-associated nephropathy, a leading cause of KTx failure, affecting 1–10% of recipients [20].

Peritoneal dialysis is a convenient kidney replacement available for use at home, but may cause chronic inflammation, Th1/Th2 imbalance, and related pathologies that may be triggered by repeated episodes of infection [21]. In peritoneal biopsies from dialysis patients, IL-17A activation was found mainly in inflamed areas and was absent in healthy peritonea. Cells expressing IL-17A included lymphocytes, especially CD4-positive lymphocytes and the gamma delta (γδ) subpopulation of T lymphocytes [22]. IL-17 is an important cytokine secreted by Th17 and shows increased local expression in the event of graft rejection. Moreover, increased Th17 cell infiltration was significantly associated with incomplete recovery, recurrent T cell-mediated kidney rejection (TCMR), steroid-resistant rejection, and lower graft survival after rejection [23]. Elevated IL-17A concentrations were found in patients on long-term peritoneal dialysis [22]. The pro-inflammatory effects of the IL-17 family of cytokines, such as IL-17A and IL-17F, are mediated by the activation of their IL-17RA/IL-17RC receptors, which are expressed in most kidney cells and are involved in the activation of many pro-inflammatory and pro-fibrotic pathways [24,25]. Additionally, genetic variations within IL-17 and IL-17 receptor genes contribute to the risk of developing kidney allograft failure and graft loss [26,27,28,29]. However, some IL-17 genotypes and alleles may be associated with a lower risk of acute rejection and better graft survival [30].

Recent studies have associated microRNAs with pathological processes after KTx such as T cell- (TCR) or antibody-mediated rejection (AMR) and delayed graft function [31,32]. MicroRNAs are short, endogenous, non-coding RNAs that inhibit gene expression and are involved in various cellular processes [33,34,35]. For example, miR-101-3p, miR-127-3p, miR-210-3p, miR-126-3p, miR-26b-5p, miR-29a-3p, miR-142-3p, miR-155, miR-223, miR-142-5p, miR-146a-5p, miR-27a-3p, miR-650, miR-93-3p, miR-10a-5p, miR-10b, miR-15b, and miR-16 have been shown to be altered in serum samples from patients with an acute kidney injury (AKI) [36,37]. The miR-146 family (miR-146a and miR-146b) is homologous and only two nucleotides differ in the 3′ region. Their coding genes are located in humans on chromosomes 10 (10q24.32) and 5 (5q33.3) [38]. In the physiological state, miR-146a-5p expression is restricted to immune cells and negatively inhibits the innate and adaptive immune response by regulating certain adapters or transcription factors, including, e.g., the signal transducer and activator of transcription 1 (STAT1) [39]. Moreover, miR-146a-5p influences gene expression through multiple signaling pathways such as TNF, NF-κB, and MEK-1/2, and JNK-1/2 [40]. The expression levels of miR-146a-5p were increased in patients with kidney diseases, with focal segmental glomerulosclerosis, and membranoproliferative glomerulonephritis [41]. Additionally, the expression of miR-146a-5p was examined in renal tissue and peripheral blood during delayed graft function. It was significantly increased in the renal biopsies of patients with delayed graft functions compared to stable recipients and patients with acute rejection, and a similar trend was found in peripheral blood samples [41]. In recipients who received kidneys from deceased donors, the warm ischemia time was longer than in recipients who received organs from living donors, and the former had higher urinary miR-146a-5p levels than that of the latter type of recipients. This study suggests that urinary miR-146a-5p expression is positively associated with the degree of ischemia/reperfusion injury (which is the main cause of AKI) [42]. miRNA-146a has been shown to have a high diagnostic value in intensive care unit patients and shows a strong and significant downregulation during early AKI [41].

In the present study, eGFR was analyzed as a marker of kidney function in combination with other immunological parameters related to the Th17 cytokine profile as well as the expression and polymorphism of the *miR-146a-5p* molecule. We hypothesize that, despite the unfavorable outcome for the recipient, some IL-17/IL-17R genotypes and alleles may have a beneficial effect on graft function in the initial period after KTx. Our study also suggests that the miR-146a-5p expression in serum might serve as a potential biomarker for the long-term outcome post-KTx.

## 2. Materials and Methods

### 2.1. Patients and Healthy Controls

The study included 108 patients after dialysis, 37% female and 63% male, with a mean age of 51 years (median 20–73 years) subjected to KTx. After KTx, they were treated at the Department of Nephrology and Transplantation Medicine of the Wroclaw Medical University (Wroclaw, Poland). Blood samples for genetic testing were collected from patients placed on the transplant waiting list, after obtaining their informed consent. The study was approved by the Bioethical Committee of the Wroclaw Medical University and performed in accordance with the World Medical Association Declaration of Helsinki. Additionally, 125 healthy blood donors were used as a control group in the genotyping part of the study. The control group was recruited from the Regional Transfusion Center and Blood Bank in Wrocław (Poland). These people were not related to the recipients.

A subgroup of 89 patients received kidney allografts from a deceased person, whereas the remaining 19 patients received a kidney transplant from living donors. The patients were followed for up to 109 months after KTx. This is a retrospective study, and anti-HLA class I and anti-HLA class II donor-specific antibodies (DSAs) were determined only in highly sensitized and second transplants patients at the time of sample collection (n = 19). The characteristics of the study group are presented in Table 1.

### 2.2. Genotyping

Genomic DNA was isolated from the peripheral blood of pre-KTx dialysis patients and healthy individuals using the Maxwell 16 Blood DNA Purification Kit (Promega Corp., Madison, WI, USA) following the manufacturer’s protocol. A DeNovix DS-11 spectrophotometer (DeNovix Inc., Wilmington, DE, USA) was used to measure the DNA concentration and to assess its purity. Subsequently, the extracted DNA was stored at −20 °C until further use.

The selection of the studied single nucleotide polymorphisms (SNPs) within genes coding for *IL-17A* (rs2275913; G/A), *IL-17F* (rs763780; A/G), *IL-17RA* (rs4819554; A/G), *IL-17RC* (rs708567; G/A), and *miR-146a-5p* (rs2910164; G/C) was based on the results of the SNP Function Prediction tool available on the website of the National Institute of Environmental Health Sciences (NCBI Database), as well as other auxiliary databases (https://snpinfo.niehs.nih.gov/snpinfo/snpfunc.html (accessed on 20 January 2024); https://www.ncbi.nlm.nih.gov/snp/ (accessed on 20 January 2024); https://www.ensembl.org/index.html (accessed on 9 January 2024)). The following criteria were used: a minor allele frequency in Caucasians above 10%, a change in RNA and/or amino acid chain, potential splicing site and/or miRNA-binding site.

The genotyping of selected SNPs was performed using LightSNiP assays (TIB MOLBIOL, Berlin, Germany) on the LightCycler 480 Real-Time PCR Instrument (Roche Diagnostics, Basel, Switzerland).

### 2.3. IL-17A and IL-17F Serum Levels

As part of routine tests before KTx (48 h after dialysis, approximately 11 months before KTx), serum was collected. A sandwich enzyme-linked immunosorbent assay (ELISA) was used to assess IL-17A (IL-17 Quantikine ELISA Kit (D1700), R&D Systems, Minneapolis, MN, USA) and IL-17F (Human IL-17F DuoSet ELISA (DY1335B), R&D Systems, USA) concentrations in the serum samples of 77 patients. Measurements were performed in duplicate following the manufacturers’ protocols. Subsequently, absorbance was measured in a Sunrise microplate reader with Magellan analysis software (version 7.2, Tecan Trading AG, Männedorf, Switzerland). The standard curve allowed for the measurement of IL-17A concentrations ranging from 31.2 to 2000 pg/mL and for IL-17F concentrations ranging from 12.5 to 800 pg/mL.

### 2.4. miR-146a-5p Expression

For the analysis of miRNA-146a expression, RNA was isolated from the serum of 12 patients before KTx and from 16 healthy controls with the use of Nucleospin miRNA Plasma (MACHEREY–NAGEL GmbH&Co.KG, Düren, Germany). Reverse transcription was conducted using the TaqMan MicroRNA Reverse Transcription Kit (Applied Biosystems, Life Technologies, Foster City, CA, USA), in accordance with the manufacturer’s protocol. The reaction was carried out in a SimpliAmpTM Thermal Cycler (Applied Biosystems, Life Technologies, Foster City, CA, USA) at 16 °C for 30 min, 42 °C for 30 min, and 85 °C for 5 min. The product of reverse transcription was stored at −20 °C until its further use. The expression of miR-146a-5p was analyzed using Real-Time PCR. The reaction was performed on a ViiaTM 7 Real-Time PCR System (Applied Biosystems, Life Technologies, Foster City, CA, USA) using the TaqMan microRNA Assay. Primers for human miR-146a-5p and U6 together with TaqMan Universal PCR Master Mix II, no UNG (Applied Biosystems, Life Technologies, Foster City, CA, USA) were used. miR-146a-5p expression was normalized to U6, which served as an endogenous, small, nuclear RNA control (TaqMan MicroRNA Assays, Applied Biosystems). All reactions were carried out in duplicate. Expression was calculated using the ^ΔΔ^Ct method.

### 2.5. Statistical Analysis

The Fisher’s exact test was used to test the null hypothesis that there was no difference in the allele and genotype frequency of the analyzed SNPs between Ktx patients and healthy individuals (calculated using the online tool http://vassarstats.net/, accessed on 6 January 2024). Data normality was assessed using the Shapiro–Wilk test. In case of a deviation from the normal distribution, the data was analyzed with the non-parametric Mann–Whitney *U*-test. All of these calculations were performed using GraphPad Prism software (GraphPad Software, La Jolla, CA, USA, version 8.0.1) and the Real Statistics Resource Pack for Microsoft Excel 2019 (version 16.0.10402.20023, Microsoft, Redmond, WA, USA). The analysis was performed in RStudio v.4.2 (RStudio, PBC, Boston, MA, USA). *p*-Values < 0.05 were considered statistically significant, while those between 0.05 and 0.10 were indicative of a trend.

## 3. Results

### 3.1. Recipient Characteristics

The characteristics of the study group before and after KTx are presented in Table 1. In our study, the estimated glomerular filtration rate (eGFR) was used as a parameter reflecting recipient graft function, which is a predictor of graft survival. We observed statistically significant differences in the eGFR levels between the first and twelfth month after KTx (*p* = 0.0378; Table 1). Additionally, a significant association was observed between HLA donor–recipient compatibility and the absence or presence of DSA. In our study group, only seven KTx patients with an HLA-A mismatch (one or two) produced DSA (*p* = 0.0512).

### 3.2. SNP Distribution in Patients and Controls

We analyzed the allele and genotype distribution of the SNPs tested within genes coding for *IL-17A*, *IL-17F*, their receptors *IL-17RA* and *IL-17RC,* and *miR-146a-5p*. The results are shown in Table 2. We did not observe any relationship between the selected SNPs in genes coding for *IL-17A*, *IL-17RA, IL-17RC,* and *miR-146a-5p* and kidney disease risk, as their alleles/genotypes segregated similarly in patients and controls.

**Figure 1:**
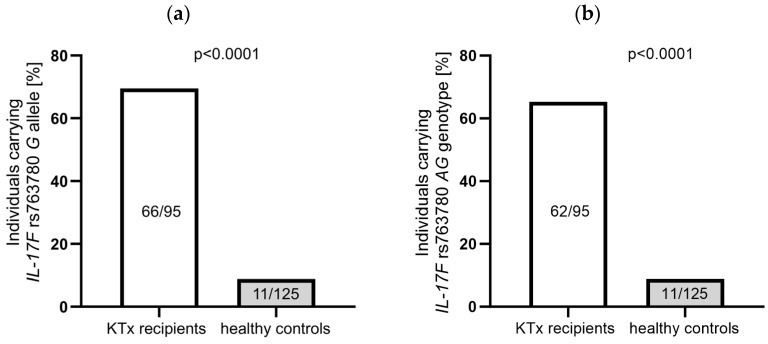
The differences in the frequency of the *IL-17F* rs763780 *G* allele (**a**) and the *AG* genotype (**b**) between KTx patients and healthy controls. Statistical analysis was performed using Fisher’s exact test (**a**,**b**).

However, KTx recipients were more often characterized by the presence of the *IL-17F* rs763780 *G* allele than healthy individuals were (66/95 vs. 11/125, OR = 23.5862, 95%CI 11.0595–50.3014, *p* < 0.0001, Figure 1a). Additionally, the presence of the *IL-17F* heterozygous genotype was also more common in a group of patients than in healthy subjects (62/95 vs. 11/125, OR = 0.0514, 95%CI 0.0243–0.1086, *p* < 0.0001; Figure 1b). These results suggest that allele *G* rs763780 *IL-17F* may affect the development of kidney diseases.

### 3.3. IL-17A and IL-17F Polymorphism and Serum Levels, and miR-146a-5p Polymorphism and Expression

**Figure 2:**
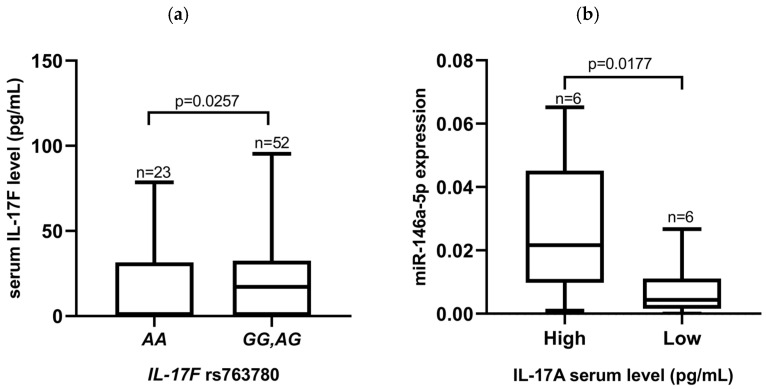
Relationships between IL-17A/IL-17F serum levels, gene polymorphisms, and miR-146a-5p expression. (**a**) The *IL-17F* rs763780 *G* allele is associated with an increased IL-17 concentration in the serum of patients; (**b**) a higher expression of miR-146a-5p is associated with an increased IL-17A serum concentration. No significant association was observed between the IL-17A variant and the IL-17A serum level or the IL-17A concentration and miR-146a-5p expression (not shown). Statistical analysis was performed using a Mann–Whitney test (**a**) and an unpaired *t*-test (**b**).

The *IL-17F* (rs763780) genotypes in recipients have been found to be associated with TCMR. In patients carrying the *G* allele (*AG* and *GG* genotypes), no TCMR was observed, while in the group of patients with the *AA* genotype, four individuals developed TCMR (OR = 9.6, 95%CI 1.0215–90.2181, *p* = 0.0357). Moreover, this polymorphism was found to be related to the IL-17 concentration in patients’ serum. The serum level of IL-17F was higher for patients carrying *AG* and *GG* genotypes (*G* allele) compared to *AA* homozygotes (20.45 vs. 11.53, *p* = 0.0257, Figure 2a). No associations between IL-17F serum levels and the expression of miR-146a-5p were observed.

Conversely, the IL-17A concentrations were not associated with different *IL-17A* rs2275913 genotypes, although a regulatory role of miR-146a-5p expression on IL-17A levels was demonstrated. For the purpose of further analyses, we set the cut-off level of IL-17A at 20.08 pg/mL, and defined levels above that value as being high. All values below this level were defined as being low. Higher miRNA-146a expression was more frequently detected in patients with increased IL-17A serum concentrations as compared to patients with a lower level of this cytokine (0.0319 vs. 0.0064, *p* = 0.0177; Figure 2b). Additionally, we observed that nine patients carrying the *IL-17A* rs2275913 *GG* genotype were more likely to die before the KTx procedure than four patients with the *A* allele were (OR = 0.2400, 95%CI 0.0676 0.8524, *p* = 0.0307). Moreover, six KTx patients with the *miR-146a-5p* rs2910164 *C* allele more frequently developed TCMR or AMR than did two patients with the *GG* genotype (OR = 5.3793, 95%CI 1.0192–28.3922, *p* = 0.0531).

**Figure 3:**
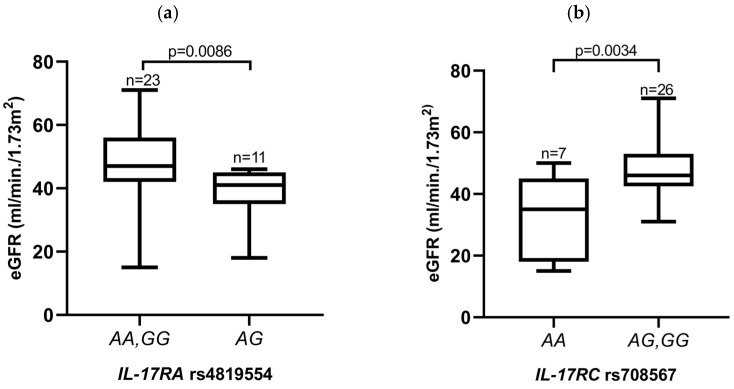
The relationships between *IL-17RA* (rs4819554) and *IL-17RC* (rs708567) recipient genotypes and eGFR at one month after kidney transplantation. (**a**) A significant association with a higher eGFR was detected for the *IL-17RA* (rs4819554) homozygous genotypes and (**b**) the *IL-17RC* (rs708567) *G* allele. eGFR was calculated using the MDRD formula. Statistical analysis was performed using a Mann–Whitney test (**a**) and an unpaired *t*-test (**b**).

eGFR levels did not seem to be associated with either *IL-17A* or *IL-17F* SNPs. Interestingly, significant differences were detected when the SNPs located within genes coding for IL-17 receptors were considered. Recipients with the homozygous *IL-17RA* (rs4819554) genotypes *AA* or *GG* showed higher eGFR values compared to *AG* heterozygotes (47.17 vs. 38.73, *p* = 0.0086; Figure 3a). Regarding the *IL-17RC* (rs708567) SNP, a higher eGFR was seen in patients carrying the *G* allele compared to *AA* homozygous patients (47.62 vs. 34.29, *p* = 0.0034; Figure 3b).

## 4. Discussion

Th17 lymphocytes together with Treg cells are strongly implicated in kidney transplant outcomes. The imbalance between these two lymphocyte populations, associated with elevated inflammatory cytokine production, may significantly affect kidney graft survival [11,12]. The genetic variability within genes coding for immunomodulatory cytokines may be associated with protein production and transplant outcomes. Our previous studies in patients with autoimmune diseases documented the important role of the SNPs located within genes coding for *IL-17A*, *IL-17F* cytokines as well as their *IL-17RA* and *IL-17RC* receptors [43,44].

Despite significant progress in understanding the pathophysiology of kidney disease, current therapies continue to be limited and often ineffective. Several immunosuppressive therapies targeting Th17, Tregs, and Th17/Tregs are available, but these therapies have some limitations [45,46,47]. The most current and most promising strategy is the regulation of the metabolic reprogramming of Treg cells. This procedure involves isolating autologous Treg cells and then multiplying them in sterile conditions. During this process, Treg cell function and metabolism can be modified with drugs or the metabolite substrate composition can be changed. After the expansion process, Treg cells can be returned to the patient [48].

The interaction between recipient and donor risk factors makes the prognosis of KTx recipients one of the most important topics in modern transplantology. For example, an HLA-DQ mismatch is associated with a higher one-year risk of acute rejection and lower graft survival in living and deceased donor kidney transplants [49]. More recent studies on the MHC class I MICA gene also documented the association between donor–recipient MICA mismatches and decreased kidney graft survival [50]. Donor-specific antibodies (DSAs) are antibodies directed against specific HLA antigens of the donor and have become an established biomarker predicting AMR [51]. De novo developed DSAs after KTx are associated with late AMR, chronic antibody-mediated rejection, and transplant glomerulopathy [52,53]. In the present study, we observed no significant associations between HLA mismatch and KTx outcome, although we showed an association between HLA mismatch and DSAs. We noticed that KTx patients with an HLA-A mismatch (one or two) produced DSAs. Iwahara et al. showed an increase in Th1 and Th17 responses in patients with DSAs. They suggest that Th1 and Th17 responses may be activated in KTx recipients by DSAs [54].

The analysis of biomarkers such as *IL-17A* and *IL-17F* may be interesting in the context of genetic heterogeneity, pre-KTx serum levels and their relationship with miR-146a-5p expression levels, as well as their impact on eGFR, especially in groups of end-stage dialysis patients. The importance of Th17 lymphocytes was shown by Mortazavi et al., who measured the mRNA and protein expression of Treg and Th17-related cytokines in the cultured PBMCs of patients at different time points after KTx [55]. They observed the increased expression of IL-10 and the decreased expression of IL-6, IL-17, and IL-23 in the transplant groups during the follow-up. There were no significant differences in IL-17 and IL-23 serum levels during the 6 to 36 months range and over 3 years after KTx. On the other hand, IL-6, IL-17, and IL-23 cytokine levels were significantly lower during the early observation period from 1 to up to 6 months after KTx compared to later time periods [55]. We examined the IL-17A and IL-17F serum concentrations as well as the SNPs within the genes of both cytokines and their receptors. We hypothesized that the early posttransplant period may play a major role in achieving immune tolerance with a low complication rate also in the context of Th17 cytokines, both in their polymorphism and expression levels. In the present study, the polymorphisms within genes coding for *IL-17RA* and *IL-17RC* showed interesting associations with the eGFR levels in KTx patients one month after transplantation. It appeared that higher eGFRs were detected in patients carrying the homozygous genotype *IL-17RA* (rs4819554), and in patients carrying the *IL-17RC* (rs708567) *G* allele (Figure 3a,b). Please note that the *IL-17F* (rs763780) SNP deserves special attention due to its potential association with a predisposition to kidney diseases (differences in the genotype distribution between patients and healthy controls; Figure 1a,b and Table 2), as well as with the serum IL-17F concentration in transplant recipients (with *G* allele carriers being higher IL-17F producers; Figure 2a). Additionally, in recipients with the *IL-17F* (rs763780) *G* allele, TCMR was observed less frequently.

In a group of Spanish patients, Coto et al. found a significantly higher frequency of *IL-17RA* rs4819554 *AA* homozygotes among individuals with an eGFR < 60 mL/min/1.73 m^2^, which was an effect independent of the presence of disease. Additionally, they showed that the *IL-17RA A* allele has been associated with the risk of developing ESRD, and was also linked to the increased expression of the IL-17RA protein and higher levels of Th17 cell subsets [27]. In our study, *IL-17RA* (rs4819554) heterozygous recipients were characterized by lower posttransplant eGFR values. Romanowski et al. showed an association of the *IL-17A* rs2275913 *GG* genotype with significantly impaired long-term kidney allograft function, as well as an association of the *IL-17F* rs11465553 *GA* heterozygosity with a higher risk of graft function loss and a return to dialysis after KTx [28]. On the other hand, the *IL-17F AA* genotype (7489 A/G) and the *A* allele might be associated with a lower risk of acute rejection and with better graft survival in Tunisian recipients [30]. Moreover, research conducted by Domanski et al. suggests a possible association between the *IL-17A* and *IL-17F* gene polymorphisms and the chronic histopathological changes detected in kidney biopsies after transplantation [56]. It appeared that tubular atrophy and interstitial fibrosis were more severe among individuals with the *IL-17F* rs763780 *C* allele while *IL-17A* rs2275913 polymorphism was found to be associated with a higher grade of tubulitis among patients with the *A* allele, and with a higher grade of arteriolar hyaline thickening and a mesangial matrix increase among patients carrying the *G* allele [56].

The functional SNP rs2910164 C>G of *miR-146a* is located in pre-miR-146a. Previous studies have shown that rs2910164 can affect the expression level of mature miR-146a [57,58]. *miR-146a* rs2910164 has been shown to influence miR-146a expression levels and may correlate with susceptibility and progression in patients with primary IgA nephropathy (IgAN) [59]. Yang et al. observed that IgAN patients carrying the ***C*** allele were more likely to develop the disease at a younger age than patients with the ***G*** allele of rs2910164 [60]. In this study, we observed that KTx patients with the *miR-146a-5p* rs2910164 *C* allele more frequently developed TCMR or AMR than did patients with the *GG* genotype. Similar to our results, Boštjančič et al. found that the *CC* genotype and the ***C*** and ***G*** alleles of *miR-146a* were associated with an increased risk of rejection when comparing rejected and non-rejected KTx patients [61]. Additionally, we found that the IL-17A concentration appeared to be associated with *miR-146a-5p* expression (Figure 2b). We observed that an elevated serum level of IL-17A in recipients before KTx was associated with higher *miR-146a-5p* expression. Also, our previous study involving patients with rheumatoid arthritis showed potential associations between the *miR-146a-5p* (rs2910164, G>C) and NFκB1 (rs28362491, ins/del ATTG) polymorphisms and miR-146a-5p expression in patients’ sera that were analyzed in relation to the clinical outcome of the treatment as well as disease susceptibility [62]. Additionally, the *miR-146a-5p* rs2910164 polymorphism was shown to alter miR-146a-5p expression and was found to be significantly associated with CTLA and TLR4 gene regulation in an African American cohort of KTx recipients [63]. Other studies showed that the expression of miR-146a-5p in biopsy samples was significantly increased in a group of patients with delayed graft function versus stable patients and patients with acute rejection [41]. The results of Li et al. indicated that IL-17A-induced miR-146a-5p may regulate the inflammatory response during the infection of *H. pylori* in an NFκB1 manner [64].

The current scientific literature clearly shows that miRNAs are the most promising among transcriptomic biomarkers because circulating miRNAs can reflect the physiological or pathological status of patients [65]. Importantly, miRNAs can be readily detected not only in tissue samples but also in body fluids such as blood and urine [42]. Their ability to regulate biological processes and their stability in body fluids make them excellent candidates as non-invasive biomarkers and potential therapeutic targets for kidney graft failure [42]. Technically, miRNAs may have optimal biochemical properties to become easily accessible indicators. These small transcripts are very stable, have a long half-life in biological samples, their analysis does not require special procedures, and they can be applied to currently available samples. miRNAs can be quantified with a relatively low cost, high sensitivity, and high specificity using standard techniques already used in clinical laboratories such as quantitative PCRs. The lack of consensus on the methodologies used to quantify miRNAs is one of the main factors limiting the application of these transcripts [66]. Other pre-analytical and analytical aspects should be considered, including the significant technical variability associated with RNA isolation, the lack of robust internal controls, and the impact of factors such as age, gender, and drug therapies on miRNA levels [66,67]. Harmonized methods for miRNA isolation and quantification and the use of standard operating procedures are crucial to improve the reproducibility of independent studies [66].

Our results suggest that the pre-transplant characteristics of the recipient, including the assessment of IL-17A and IL-17F serum levels as well as the genetic variants located within IL-17A/F and IL-17RA/RC encoding genes, may be helpful and play a prognostic role in transplant success. The specific genetic profiles of the recipient and the associated serum protein concentrations may facilitate better transplant outcomes reflected by higher eGFR values, especially in the early post-KTx period, which may further translate into a beneficial effect in the long-term follow-up after KTx.

We are aware that our work has certain limitations. To distinguish the background of post-KTx complications from stable graft function, we used the analysis of biomarkers derived from the blood and serum of patients before kidney transplantation in the context of the dynamics of eGFR changes. We relied on a wide spectrum of clinical data before and after KTx at 1, 3, 12, and 109 months. Our limitations resulted from the small amount of materials from patients after transplantation, as well as the lack of another source of materials from patients, especially after KTx. Future studies should include more diverse biological materials, including urine or biopsy samples, with an expanded panel of biomarkers that can be used to assess the stable function of the transplant. Additionally, to distinguish the background of complications after KTx from stable graft function, the number of patients should be increased. Future studies should determine a broad panel of biomarkers using liquid biopsy (bypassing blood and serum) samples, which will require the availability of larger amounts of biological materials and the use of new techniques.

Analyzing circulating IL-17 levels and combining these results with genetic studies and miRNA-mediated regulatory mechanisms in kidney transplant patients may help us understand the immunological mechanisms associated with inflammation in patients with kidney disease. Moreover, these parameters may translate into an improvement in the effect of transplantation therapy.

## 5. Conclusions

Our study demonstrated significant relationships between the polymorphisms of the IL-17A/IL-17F cytokine genes, their levels in serum, and the polymorphism and expression of miR-146a-5p analyzed before transplantation in kidney recipients. Firstly, we documented that the heterozygous genotype of *IL-17F* rs763780 appeared to influence the development of kidney diseases. Secondly, recipients with the *IL-17F* rs763780 *AA* genotype were characterized by having lower IL17F serum levels before KTx, and this genotype was also associated with T cell-mediated renal transplant rejection (TCMR) similar to patients with the *C* allele of *miRNA-146a* rs2910164. Thirdly, recipients with the *IL17A* rs2275913 *GG* genotype died more often before KTx than did patients with the *A* allele.

## Figures and Tables

**Table 1:** The characteristics of the study group before and after kidney transplantation.

Kidney Recipients	N = 108
Mean recipient age at KTx (median, range)	48 years (51; 20–73 years)
Mean donor age (median, range)	45 years (46; 16–76 years)
Recipient gender	
Male, n (%)	N = 68 (63%)
Female, n (%)	N = 40 (37%)
Donor gender	
Male, n (%)	N = 57 (53%)
Female, n (%)	N = 36 (33%)
Not known (%)	N = 15 (14%)
Donor–recipient sex matched pairs	
Male–Male, n (%)	N = 23 (72%)
Female–Female, n (%)	N = 9 (28%)
Donor–recipient sex mismatched pairs,	
Female–Male, n (%)	N = 12 (44%)
Male–Female, n (%)	N = 15 (56%)
Mean recipient BMI (kg/m^2^), (median, range)	25.60 (25.53, 16.22–36.39)
Dialysis	
Peritoneal	N = 6
Hemodialysis	N = 102
Dialysis duration (years), (median, range)	4 years (3 years, 1–17 years)
Number of recipients with panel reactive antibodies (PRA) maximum > 0%	N = 24 (3–99%)
Donor–recipient HLA incompatibility	
HLA-A	N = 73
HLA-B	N = 80
HLA-DR	N = 62
Death before KTx	N = 11 (10%)
Induction of immunosuppression ^†^	
Basiliximab	N = 10 (13%)
ATG/Thymoglobulin	N = 2 (3%)
Maintenance immunosuppression ^†^	
Tac/MMF/steroids	N = 60 (91%)
CsA/MMF/steroids	N = 6 (9%)
Mean eGFR [mL/min/1.73 m^2^]	
1 month post KTx	42.85 (median 44; range: 15–71) ^(^*^) (^**^)^
3 month post KTx	46.11 (median 48; range: 7–91)
1 year post KTx	47.31 (median 51.50; range 6–94) ^(^*^)^
>12 to <66 month post KTx	44.12 (median 50; range: 6–85)
>66 to 109 month post KTx	49.28 (median 53.50; range 8–94) ^(^**^)^
Transplant outcome	
Death after KTx	N = 4 (4%)
Delayed graft function	N = 10 (9%)
T cell-mediated rejection (TCMR)	N = 11 (10%)
Antibody-mediated rejection (AMR)	N = 4 (4%)
Graft loss during 108 months of observation	N = 12 (11%)

Abbreviations: KTx: kidney transplantation; BMI: body mass index; Tac: tacrolimus; MMF: mycophenolate mofetil or sodium; CsA: cyclosporine A; eGFR: estimated glomerular filtration rate, MDRD formula. ^(^*^)^ *p* = 0.0378; ^(^**^)^ *p* = 0.0363, ^†^—data available for 66 recipients.

**Table 2:** The distribution of *IL-17A/-F*, *IL17-RA/-RC*, and *miR-146a-5p* genotypes in kidney transplant recipients and healthy individuals. Statistical analysis was performed using Fisher’s exact test.

Gene	KTx Patients		Controls		Model OR (95%Cl); *p*-Value			
	n	%	n	%	Dominant	Recessive	Codominant	Overdominant
*IL-17A* rs2275913	N = 91		N = 125		*AA* + *GA* vs. *GG* OR = 0.6375 (0.3620–1.1226); *p* = 0.1477	*AA* vs. *GA* + *GG* OR = 0.6481 (0.2876–1.4607); *p* = 0.3252	*AA* vs. *GA* vs. *GG* OR = 1.4827 (0.8209–2.6780); *p* = 0.2282 OR = 1.9474 (0.8046–4.7129); *p* = 0.1922	*AA* + *GG* vs. GA OR = 1.2339 (0.7184–2.1194); *p* = 0.4916
*GG*	37	40.7	38	30.4				
*GA*	44	48.3	67	53.6				
*AA*	10	11.0	20	16.0				
*IL-17F* rs763780	N = 95		N = 125					
*AA*	29	30.5	114	91.2	*GG* + *AG* vs. *AA* OR = 23.5862 (11.0595–50.3014); *p* < 0.0001	*GG* vs. *AG* + *AA* OR = 5.4945 (0.6040–49.9827); *p* = 0.1675	*GG* vs. *AG* vs. *AA* OR = 0.0451 (0.0211–0.0965); *p* < 0.0001 OR = 0.0636 (0.0068–0.5908); *p* = 0.0089	*GG* + *AA* vs. *AG* OR = 0.0514 (0.0243–0.1086); *p* < 0.0001
*AG*	62	65.3	11	8.8				
*GG*	4	4.2	0	0				
*IL-17RA* rs4819554	N = 95		N = 112					
*AA*	56	58.9	66	58.9	*GG* + *AG* vs. *AA* OR = 0.9992 (0.5732–1.7418); *p* = 0.5555	*GG* vs. *AG* + *AA* OR = 0.6593 (0.1870–2.3248); *p* = 0.5535	*GG* vs. *AG* vs. *AA* OR = 0.9455 (0.5300–1.6867); *p* = 0.8832 OR = 1.4848 (0.4132–5.3355); *p* = 0.7535	*GG* + *AA* vs. *AG* OR = 0.9159 (0.5179–1.6195); *p* = 0.7730
*AG*	35	36.8	39	34.8				
*GG*	4	4.2	7	6.3				
*IL-17RC* rs708567	N = 92		N = 120					
*AA*	26	28.3	42	35.0	*GG* + *AG* vs. *AA* OR = 0.9616 (0.5589–1.6542); *p* = 0.8909	*GG* vs. *AG* + *AA* OR = 1.4085 (0.7157–2.7719); *p* = 0.3852	*GG* vs. *AG* vs. *AA* OR = 0.7979 (0.4270–1.4909); *p* = 0.5277 OR = 0.6190 (0.2844–1.3476); *p* = 0.2407	*GG* + *AA* vs. *AG* OR = 0.9616 (0.5589–1.6542); *p* = 0.8909
*AG*	45	48.9	57	47.5				
*GG*	21	22.8	21	17.5				
*miR-146a-5p* rs2910164	N = 89		N = 96					
*GG*	54	60.7	63	65.6	*CC* + *GC* vs. *AA* OR = 1.2374 (0.6801–2.2514); *p* = 0.5425	*CC* vs. *GC + AA* OR = 0.8023 (0.1745–3.6889); *p* = 0.5420	*CC* vs. *GC* vs. *AA* OR = 0.7768 (0.4178–1.4443); *p* = 0.4346 OR = 1.1429 (0.2449–5.3335); *p* = 0.5898	*CC + AA* vs. *GC* OR = 0.7710 (0.4172–1.4249); *p* = 0.4366
*GC*	32	35.6	29	30.2				
*CC*	3	3.7	4	4.2				

## Data Availability

The original data presented in the study are included in the article further inquiries can be directed to the corresponding author.
